# Antimicrobials Are a Photodynamic Inactivation Adjuvant for the Eradication of Extensively Drug-Resistant *Acinetobacter baumannii*

**DOI:** 10.3389/fmicb.2019.00229

**Published:** 2019-02-13

**Authors:** Agata Wozniak, Aleksandra Rapacka-Zdonczyk, Nico T. Mutters, Mariusz Grinholc

**Affiliations:** ^1^Laboratory of Molecular Diagnostics, Department of Biotechnology, Intercollegiate Faculty of Biotechnology, University of Gdańsk and Medical University of Gdańsk, Gdańsk, Poland; ^2^Institute for Infection Prevention and Hospital Epidemiology, Medical Center – Faculty of Medicine, University of Freiburg, Freiburg, Germany

**Keywords:** *Acinetobacter baumannii*, antimicrobials, antimicrobial blue light, photodynamic inactivation, rose bengal, synergy

## Abstract

The worldwide emergence of extensively drug resistant (XDR) *Acinetobacter baumannii* has reduced the number of antimicrobials that exert high bactericidal activity against this pathogen. This is the reason why many scientists are focusing on investigations concerning novel non-antibiotic strategies such as antimicrobial photodynamic inactivation (aPDI) or the use of antimicrobial blue light (aBL). Therefore, the aim of the current study was to screen for antimicrobial synergies of routinely used antibiotics and phototherapies, including both aPDI involving exogenously administered photosensitizing molecules, namely, rose bengal, and aBL, involving excitation of endogenously produced photoactive compounds. The synergy testing was performed in accordance with antimicrobial susceptibility testing (AST) standards, including various methodological approaches, i.e., antibiotic diffusion tests, checkerboard assays, CFU counting and the evaluation of postantibiotic effects (PAEs). We report that combining antimicrobials and aPDI/aBL treatment led to a new strategy that overcomes drug resistance in XDR *A. baumannii*, rendering this pathogen susceptible to various categories of antibiotics. Sublethal aPDI/aBL treatment in the presence of sub-MIC levels of antimicrobials effectively killed *A. baumannii* expressing drug resistance to studied antibiotics when treated with only antibiotic therapy. The susceptibility of XDR *A. baumannii* to a range of antibiotics was enhanced following sublethal aPDI/aBL. Furthermore, 3′-(*p*-aminophenyl) fluorescein (APF) testing indicated that significantly increased reactive oxygen species production upon combined treatment could explain the observed synergistic activity. This result represents a conclusive example of the synergistic activity between photodynamic inactivation and clinically used antimicrobials leading to effective eradication of XDR *A. baumannii* isolates and indicates a potent novel therapeutic approach.

## Introduction

*Acinetobacter baumannii* is a threatening human pathogen. A key component of its pathogenicity is its outstanding capability to acquire resistance (Spellberg and Bonomo, 2014). Pan-drug resistant (PDR) strains that express resistance to all clinically available antibiotics are of particular concern (Valencia et al., 2009). A lack of effective antimicrobials has forced the need for the development of novel strategies to control *A. baumannii* infections. One of these approaches is antimicrobial photodynamic inactivation (aPDI) or antimicrobial blue light (aBL) (Nitzan et al., 1998; Dai et al., 2009; Cai et al., 2012; Huang et al., 2014; Yuan et al., 2017; Yang et al., 2018). These strategies exert high bactericidal efficacy toward various microbes regardless of antibiotic resistance. Moreover, the acquisition of resistance to such a method is unlikely due to the nature of the multi-targeted process (Maisch, 2015). Briefly, the mechanism of aPDI involves a combination of non-toxic photosensitizers (PSs) and visible light (Wainwright, 1998). In the presence of oxygen, light induces the formation of reactive oxygen species (ROS) by energy or electron transfer from the PS excited state; these ROS can oxidize numerous cell biomolecules, leading to bacterial killing (Grinholc et al., 2015).

The most recent discoveries concerning aPDI or aBL indicates that photoinactivation renders microbes susceptible to clinically used antimicrobial agents (Wozniak and Grinholc, 2018). Nevertheless, only limited studies aimed at analyzing the synergistic interactions between bactericidal approaches have complied with the standards imposed for scientific literature. Thus, it was barely possible to draw reliable conclusions indicating possible synergies between photoinactivation and antimicrobials. Photoinactivation of microorganisms can damage the cell envelope, genetic material or both simultaneously (Grinholc et al., 2015); thus, in the present study, we focused on analyzing whether the synergistic effect between aPDI/aBL and antimicrobials occurs and whether it is influenced by the administration of an exogenous PS such as rose bengal (RB) or thus of endogenously produced PSs such as porphyrins, which we excited with very intense blue light (aBL). Next, to provide accurate and reliable evidence that photoinactivation indeed renders microbes susceptible to antimicrobials and acts synergistically with antibiotics, in the current work, two XDR *A. baumannii* isolates together with numerous synergy testing assays guidelines from the European Committee on Antimicrobial Susceptibility Testing (EUCAST) and the Clinical Laboratory and Standards Institute (CLSI) were employed. In addition, within the current study, the interaction of aPDI/aBL with chemotherapeutic agents (from all antibiotic classes and covering all mechanisms of action) listed by the National and European Centers for Antimicrobial Susceptibility Testing (AST) was investigated.

## Materials and Methods

### Strains and Culture Conditions

*Acinetobacter baumannii* strains (no. 127, 128) were isolated from tracheal secretions and wounds from ICU patients at University Medical Center Freiburg. The profiles of resistance showed that both strains have XDR profiles (Magiorakos et al., 2012). *A. baumannii* strains were cultivated at 37°C in tryptic soy broth (TSB, bioMérieux, France) for 16 – 20 h under aerobic conditions in an orbital incubator (Innova 40, Brunswick, Germany) at 150 rpm. Moreover, two ATCC reference strains were used as a quality control for AST, i.e., *P. aeruginosa* ATCC 27853 and *Escherichia coli* ATCC 25922.

### Antimicrobial Susceptibility Testing (AST)

AST protocols followed EUCAST guidelines. The antimicrobial agents listed in Table 1 were used (Sigma-Aldrich, Germany). For AST, ETEST^TM^ (bioMérieux, France) and Sensi-Disc^TM^ (Becton Dickinson, United States) were used. Each experiment was performed in three repetitions at different time. Interpretation of the results was performed using EUCAST breakpoint tables (Version 8.1).

**Table 1:** Minimal inhibitory concentrations for antimicrobials and light conditions.

Antibiotic target	Antimicrobial category	Antibiotic	*A. baumannii* 127		*A. baumannii* 128
			
			MIC [μg/ml]^a^		MIC [μg/ml]
Protein synthesis (50S)	Lincosamides	Clindamycin	NR		
	Macrolides	Erythromycin	NR		
	Phenicols	Chloramphenicol	NR		
	Streptogamins	Quinupristin-dalfopristin	NR		
Protein synthesis (30S)	Aminoglycosides	Gentamycin	1024 (R)		1024 (R)
	Tetracyclines	Doxycycline (NR)	32		64
	Aminoglycosides	Tobramycin	≥16 (R)		≥16 (R)
	Glycylcyclines	Tigecycline	NR		
70S initiation complex	Oxazolidinones	Linezolid	NR		
Folic acid metabolism	Folate pathway inhibitors	Trimethoprim-sulfamethoxazole	512 (R)		1024 (R)
DNA-directed RNA polymerase	Ansamycins	Rifampicin	NR		
DNA gyrase	Fluoroquinolones	Ciprofloxacin	32 (R)		128 (R)
Cell-wall synthesis	Carbapenems	Imipenem	32 (R)		32 (R)
	Antipseudomonal penicillins + β-lactamase inhibitor	Piperacillin-tazobactam	512		256
	Extended-spectrum cephalosporins	Ceftazidime (NR)	512		256
	Penicillins + β-lactamase inhibitor	Ampicillin-sulbactam	128		64
	Extended-spectrum cephalosporins	Cefotaxime	NR		
	Carbapenems	Meropenem	≥16 (R)		≥ 16 (R)
	Phosphonic acid	Fosfomycin	NR		
	Monobactam	Aztreonam	NR		
Cell membrane	Polymyxins	Colistin	2 (S)		2 (S)

			**Light dose [J/cm^2^]**		

Phototherapy	aBL	Blue light (411 nm)	72.7		72.7
	aPDI	Green light (515 nm) + rose bengal (5 μM)	80		90

*NR, not recommended; S, susceptible; R, resistant. ^a^MIC values were determined within the current study.*

### Minimal Inhibitory Concentration of aBL/aPDI

The minimal inhibitory dose of aBL and aPDI was defined as the amount of light and/or PS that inhibits the growth of bacteria under experimental conditions complementary to the AST. For aBL, light with a wavelength of 411 nm was used; for aPDI treatment, the light at 515 nm and RB (5 μM) were used. Overnight bacterial cultures were adjusted in fresh MHB medium to 0.5 McFarland, 10-fold diluted and finally transferred with or without PS to a 96-well plate. For MIC estimation of aBL, light (18.2, 36.4, 54.5, 72.7, 90.9 J/cm^2^) was delivered for three independent biological samples. In the case of the MIC of aPDI, light doses of 20, 40, 60, 70, 80, 90, and 100 J/cm^2^ (70 mW/cm^2^) were delivered to three independent biological samples. After phototreatment, plates were kept in dark at 37°C for 16 – 20 h (Termaks, Norway), followed by aBL/aPDI MIC determination via measuring the medium turbidity.

### Light Sources

Illumination was performed with two light-emitting diode (LED) light sources, emitting blue (λ_max_ 411 nm, irradiance 130 mW/cm^2^, full width at half maximum (FWHM) 17 nm) and green light (λ_max_ 515 nm, irradiance 70 mW/cm^2^, FWHM 33 nm) (SecureMedia, Poland). The full characteristics of the light sources were recently published by Ogonowska et al. (2018).

### Photosensitizer

RB [4,5,6,7-tetrachloro-2′,4′,5′,7′-tetraiodofluorescein disodium salt (Sigma-Aldrich, Germany)] was dissolved in sterile water at a 1 mM concentration and kept in the dark at -20°C. For photodynamic inactivation, RB was used in two final concentrations, 5 and 10 μM.

### aBL/aPDI Treatment

Overnight bacterial cultures adjusted to 5 × 10^7^ CFU/ml were transferred to a 96-well plate alone or in combination with PS. The aPDI samples treated with RB were incubated at room temperature in the dark (15 min) and then irradiated with different light doses up to 300 J/cm^2^. The aBL samples without RB were illuminated with different light doses, with the highest value being 109.1 J/cm^2^. After illumination, a 10-μl aliquot was transferred to PBS, serially diluted and streaked horizontally on TSA plates. The control consisted of untreated bacteria. TSA plates were incubated at 37°C for 16 – 20 h, and then CFU were counted. Each experiment was performed in three independent replicates.

### Determination of the Sublethal and Lethal Doses of aBL/aPDI

Estimation of the sublethal (reduction of 0.5 – 2 log_10_ in CFU/ml) and lethal (reduction ≥ 3 log_10_ in CFU/ml) photodynamic (aBL/aPDI) treatments were assessed as the changes in survival rate of treated bacteria vs. untreated bacteria (Barry and Lasner, 1979; Dodd et al., 1997; Kohanski et al., 2010; Latimer et al., 2012; Andersson and Hughes, 2014; Amin et al., 2016; Fila et al., 2018).

### Synergy Testing

There are only a few approved methods for synergy testing that give reliable results, according to the *American Society for Microbiology*^1^: (i) disk diffusion assay; (ii) ETEST; (iii) time-kill assay [e.g., PAE (postantibiotic effect)]; and (iv) checkerboard assay (Doern, 2014). For experiments involving aPDI/aBL and antimicrobials, all of the recommended methods were used, and the survival rate of bacterial cells (CFU/ml) and the optical density (OD_580_) were determined.

#### Diffusion Assays

Bacterial cultures (5 × 10^6^ CFU/ml) were ready to use within 15 min of preparation. For experiments concerning the combined aPDI treatment, the bacterial cultures were transferred to 24-well plates with RB (1 ml per well) to receive a final PS concentration of 5 or 10 μM and then incubated for 15 min. Next, samples were treated with 515 nm light. In the case of aBL, the bacterial cultures were irradiated with 411 nm light. After phototreatment, samples were streaked on Mueller-Hinton agar plates (MHE, bioMerieux, France). Then, Sensi-Discs^TM^ and ETESTs were placed on MHE agar plates and incubated for the next 15 min at room temperature. Next, plates were incubated at 37°C for 16 h. The control consisted of bacteria not treated with aPDI/aBL. For the disk diffusion method, the synergistic effect was considered positive only when the differences in inhibition zones between the control and aBL/aPDI treatments were ≥ 2 mm. In the case of the ETEST, the synergy was defined as positive only when the MIC was 2-fold lower than the MIC for the control (untreated cells).

#### Checkerboard Assay

##### Antimicrobial blue light

The bacterial suspension (5 × 10^6^ CFU/ml) was transferred with antibiotics to a 96-well plate to achieve the following concentrations in each row: 2 × MIC, MIC, ½ MIC, ¼ MIC, and 0 × MIC, indicating the control. Next, plates were incubated in the dark for 30 min, followed by separate irradiation of each column of a 96-well plate with the following doses of aBL: 0 × MIC, 18 MIC, ¼ MIC, ½ MIC, MIC, and 2 × MIC. After exposure to aBL, plates were incubated for 16 h at 37°C. Next, the optical density was measured at 580 nm with a plate reader (Victor 1420 multilabel counter, Perkin Elmer, United States). The control group consisted of bacterial cells not treated with aBL. Each experiment was performed in three independent replicates.

##### Antimicrobial photodynamic inactivation

When aPDI was combined with antimicrobials, the rows of 96-well plates were filled with bacterial suspension combined with antibiotics in various concentrations (2 × MIC, MIC, ½ MIC, ¼ MIC, and 0 × MIC). Additionally, the wells in columns were 2-fold diluted with RB to obtain final PS concentrations of 2 × MIC, MIC, ½ MIC, ¼ MIC, 18 MIC, and 0 × MIC. The prepared plate was incubated for 30 min in the dark, and the samples were then irradiated with 515 nm light. The plates were then incubated at 37°C for 16 h, and the optical density was measured at 580 nm. The control group consisted of a bacterial suspension administered with RB but not treated with light.

The interaction of two tested compounds was defined based on the fractional inhibitory concentration index (FIC_I_), which was also defined for each tested agent separately (FIC_A_, FIC_B_). FIC_A/B_ is equal to the MIC value of drug A/B when used in combination divided by the MIC of drug A/B alone. The FIC_I_ was calculated as follows: ΣFIC_I_ = FIC_A_+FIC_B_. Regarding the guidelines, the interaction between two tested factors can be defined as synergy when FIC_I_ is ≤ 0.5 or as antagonism when FIC_I_ > 4.0 (Odds, 2003).

#### Estimation of Posttreatment Survival Rate (CFU/ml)

To estimate the changes in the survival rate of tested *A. baumannii* isolates during the checkerboard assay procedure, 10 μL of each sample was transferred to PBS 30 min after light treatment, serially diluted, streaked on TSA plates and then incubated at 37°C for 16 h. Next, the colonies were counted (CFU/ml). A synergy was confirmed when the survival rates for the combination of aBL/aPDI and antibiotic were decreased in reference to the control curve, indicating the effect of light monotherapy (aPDI/aBL).

#### Postantibiotic Effect

Overnight culture was diluted 1:20 (v/v) in TSB medium and then pretreated for 2 h with antibiotic/photosensitizer combinations as follows: (a) aPDI/aBL (MIC), (b) antibiotic (½ MIC), and (c) aPDI/aBL (MIC) + antibiotic (½ MIC). Next, PS/antibiotics were removed by two PBS washing steps. Samples a and c were transferred to a 24-well plate and singly irradiated with the MIC dose of aPDI/aBL. The control group of bacterial cells was not-treated with light or antimicrobial agents. Subsequently, 10-μl aliquot was serially diluted, streaked on TSA plates and incubated for 16 h at 37°C. Samples loaded into 24-well plates were placed in an EnVision Multilabel Plate Reader (PerkinElmer, United States), and the optical density (λ 600 nm) was measured every 40 min for 20 h (30 repetitions). Next, growth curves of bacterial cells exposed to combined treatments were compared to those of the control as well as to curves representing monotreatments (MIC aPDI/aBL, ½ MIC antibiotic). The presence of PAE (time of delayed bacterial recovery during the growth vs. time curves) indicated a possible synergistic effect. The PAE can be calculated with the following formula: (Δt) *PAE = T – C*, where *T* is the time the bacterial population requires to reach half the maximum optical density after the tested compound (e.g., antibiotic) is removed and *C* is the time required for untreated cells to reach half of the maximum absorbance (Odenholt, 2001). A synergistic effect was considered significant when the PAE parameter *Δt* ≥ 3 h and partial when 1.5 h ≤*Δt* > 2.9 h. Colony counting was necessary to establish the viable cell number in tested samples and controls.

### ROS Detection

Reactive oxygen species detection was performed using 3′-(p-aminophenyl) fluorescein (APF, Thermo Fisher Scientific, United States), which is a fluorescent indicator of hydroxyl radicals (^∙^OH). In addition, APF may also be used to detect exclusively singlet oxygen when administered with DMSO (0.1%). The protocol described by Price et al. allows the quenching of the fluorescence linked to the hydroxyl radicals (Price et al., 2009). Thus, the detection of ROS was carried out both in the absence and presence of 0.1% DMSO for combined aBL/aPDI treatment, monotreatments (aPDI/aBL) and untreated, control samples. The concentrations of APF, CST, and DOX were 10 μg/ml, 2 μg/ml, and 32 μg/ml, respectively. RB was used at a concentration of 5 μM. Combined samples were prepared in PBS in black 96-well plates and then incubated for 15 min in the dark at room temperature. Next, a 515 nm light dose of 90 J/cm^2^ was delivered. In the case of aBL, samples were exposed to a 411 nm light dose of 90.1 J/cm^2^. Fluorescence measurements were performed immediately after aPDI/aBL irradiation with an EnVision Multilabel Plate Reader (PerkinElmer, United States) at emission/excitation wavelengths of 490/515 nm.

## Results

### Experimental Workflow

**FIGURE 1:**
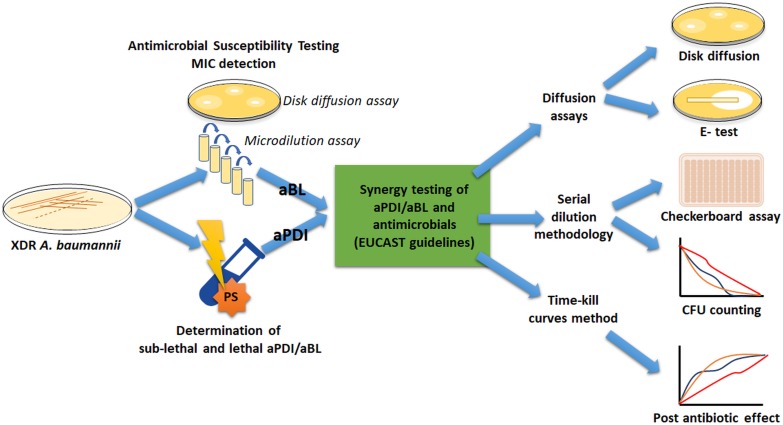
The experimental workflow.

To meet the international standards for synergy testing, numerous official AST procedures were employed to ensure that reliable conclusions were drawn; thus, we introduce a general workflow diagram to facilitate following the obtained results (Figure 1).

Two XDR *A. baumannii* isolates (nos. 127 and 128) were employed. The first stage was to characterize the drug resistance profile of *A. baumannii* isolates, followed by antimicrobial MIC evaluation. Next, overnight bacterial cultures were treated with different light doses and/or PS (i.e., RB) concentrations to determine both lethal and sublethal photo treatment conditions. The identification of sublethal doses was required because adequate synergy testing needs to be performed with living cells. Afterward, combined sublethal aPDI/aBL and sub-MIC doses of antimicrobials were investigated to find possible synergies. For proper implementation of synergy testing, various standard methodologies were used.

### Identification of Lethal and Sublethal Treatments

**FIGURE 2:**
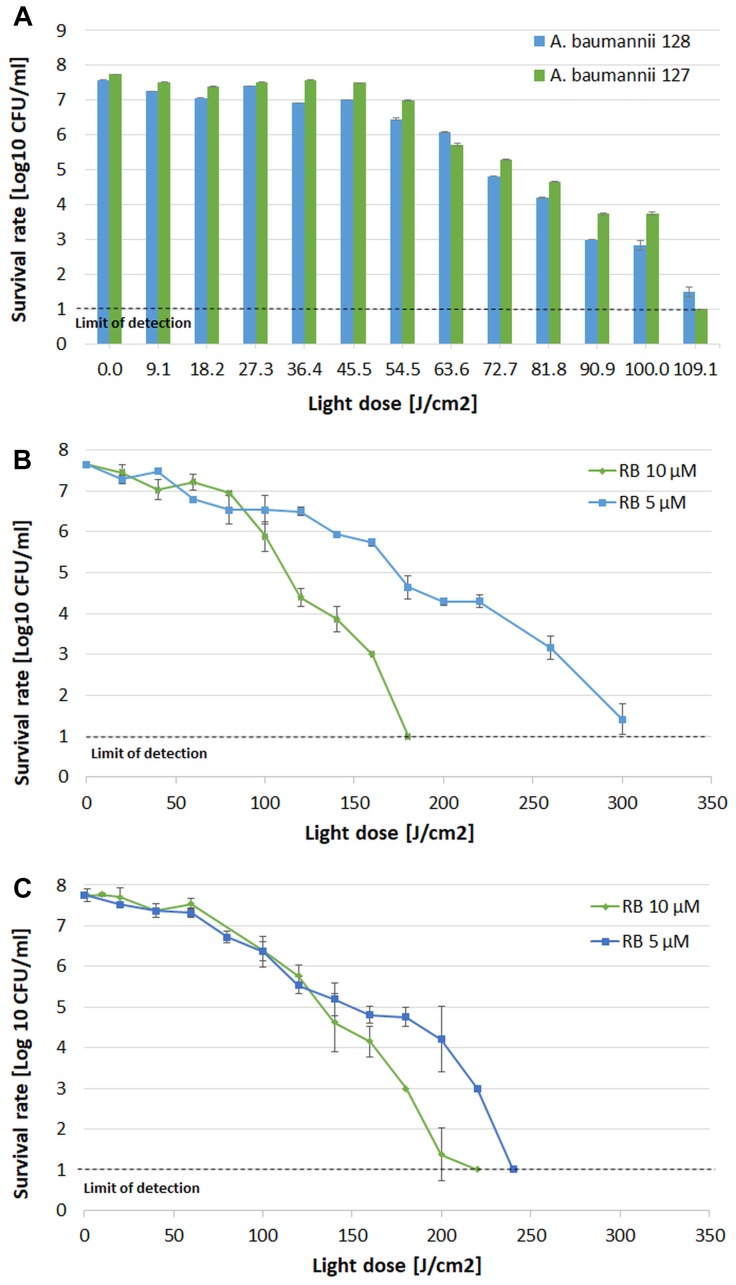
Influence of aBL and aPDI on *A. baumannii* strains. **(A)** aBL treatment of *A. baumannii* isolates. Light doses ranging from 9.1 to 109.1 J/cm^2^ (irradiance 130 mW/cm^2^, irradiation time from 79 to 952 s, λ 411 nm) were applied to two XDR strains (blue bars – strain no. 128; green bars – strain no. 127. **(B)** aPDI treatment of *A. baumannii* strain no. 128. Light doses ranging from 20 to 300 J/cm^2^ (irradiance 70 mW/cm^2^, irradiation time from 303 to 4545 s, λ 515 nm) and two rose bengal concentrations were tested (green diamonds – 10 μM; blue squares – 5 μM). **(C)** aPDI treatment of *A. baumannii* strain no. 127. Light doses ranging from 20 to 240 J/cm^2^ (irradiance 70 mW/cm^2^, irradiation time from 303 to 3636 s; λ 515 nm) and two rose bengal concentrations were tested (green diamonds – 10 μM; blue squares – 5 μM). The detection limit was 10 CFU/ml. The values are the means of three separate experiments. Values were combined by a line for better visualization of the data.

Adequate synergy testing required the preliminary characteristics of the studied *A. baumannii* isolates regarding their drug resistance profiles as well as their response to aPDI and aBL treatments. Detailed characteristics are presented in Table 1. The results indicated that both endogenously- (aBL) and exogenously administered PS (aPDI)-based phototreatments could reach high bactericidal efficacy, leading to a reduction in cell viability by ≥6 log_10_ units (Figure 2).

In the case of aBL (Figure 2A), the sublethal light dose was defined as 63.6 J/cm^2^, which reduced bacterial viability by 1.5 and 2 log_10_ (in the cases of *A. baumannii* no. 128 and 127, respectively). When considering aPDI treatment with two studied RB concentrations vs. light doses, different combinations could define sublethal conditions (Figure 2B,C). In the case of 5 μM RB, the sublethal aPDI could be defined as 160 and 100 J/cm^2^, resulting in cell viability reduction by 1.9 and 1.4 log_10_ units (in the cases of *A. baumannii* no. 128 and no. 127, respectively). When 10 μM RB was used, the sublethal light dose was defined as 100 and 120 J/cm^2^, which led to viable cell reduction by 1.7 and 2 log_10_ units (for *A. baumannii* no. 128 and 127, respectively).

### Diffusion Based Assays for Synergy Testing

First-line screening for potent synergies of antimicrobials was performed by employing diffusion-based techniques. The results indicated that in the case of both photobased treatments, the employment of sublethal aBL/aPDI conditions influenced *A. baumannii* susceptibility to numerous routinely used antimicrobials, resulting in larger growth inhibition zones (in the case of the disk-diffusion assay) and decreased MICs (for the ETEST) (Tables 2, 3). Though the impact of aPDI on *A. baumannii* drug susceptibility was observed in the cases of numerous antimicrobial agents (i.e., gentamycin, doxycycline, imipenem, ampicillin/sulbactam, and colistin), the most pronounced effect was reported for gentamycin; in this case, sublethal aPDI treatment reduced the MIC values 42-fold for *A. baumannii* no. 127 isolate from 1024 μg/ml (as stated in Table 1) to 24 μg/ml. Similar results were reported for aBL treatment (Table 3). Sublethal aBL levels resulted in larger growth inhibition zones for the *A. baumannii* no. 128 isolate (i.e., from 7.3 to 8.1 mm and from 13.7 to 14.2 mm, in the cases of imipenem and colistin, respectively) and in case of *A. baumannii* no. 127 decreased MICs (i.e., reduction in MIC from 128 to 64 μg/ml and from 48 to 32 μg/ml in the cases of doxycycline and ampicillin/sulbactam, respectively) (Table 3).

**Table 2:** Sublethal aPDI impacts on *A. baumannii* drug susceptibility.

aPDI (λ 515 nm)													
	**Antibiotic**	**Control**		**Light (5 J/cm^2^) + RB (10 μM)**		**Light (10 J/cm^2^) + RB (10 μM)**		**Light (11.25 J/cm^2^) + RB (5 μM)**		**Light (18.0 J/cm^2^) + RB (5 μM)**		**Light (22.5 J/cm^2^) + RB (5 μM)**	
		**Disk diffusion [mm]**	***E*-test [μg/ml]**	**Disk diffusion [mm]**	***E*-test [μg/ml]**	**Disk diffusion [mm]**	***E*-test [μg/ml]**	**Disk diffusion [mm]**	***E*-test [μg/ml]**	**Disk diffusion [mm]**	***E*-test [μg/ml]**	**Disk diffusion [mm]**	***E*-test [μg/ml]**

*A. baumannii* 127	GEN	6 (R)	≥256 (R)	6 (R)	≥256 (R)	6 (R)	≥256 (R)	6 (R)	≥256 (R)	6 (R)	≥256 (R)	6 (R)	24 (R)
	DOX (NR)	6	128	6	128	7.4	128	7.3	32	9.1	32	10.4	6
	SXT	6 (R)	≥32 (R)	6 (R)	≥32 (R)	6 (R)	≥32 (R)	6 (R)	≥32 (R)	6 (R)	≥32 (R)	6 (R)	≥32 (R)
	CIP	6 (R)	≥32 (R)	6 (R)	≥32 (R)	6 (R)	≥32 (R)	6 (R)	≥32 (R)	6 (R)	≥32 (R)	6 (R)	≥32 (R)
	IPM	6.8 (R)	≥32 (R)	7.9 (R)	24 (R)	10 (R)	≥32 (R)	10.2 (R)	≥32 (R)	11.6 (R)	12 (R)	12.2 (R)	8 (R)
	TZP	6	≥256	6	≥256	6	≥256	6.9	≥256	8.4	≥256	6.7	≥256
	CAZ (NR)	6	≥256	6	≥256	6	≥256	6	≥256	6	≥256	6	≥256
	SAM	6.5	48	7.1	24	7.7	32	9.7	32	10.0	12	11.2	12
	CST	14.0	0.094 (S)	14.6	0.125 (S)	14.3	0.125 (S)	13.9	0.125 (S)	16.7	0.125 (S)	18.3	0.094 (S)

	**Antibiotic**	**Control**		**Light (5 J/cm^2^) + RB (10 μM)**		**Light (10 J/cm^2^) + RB (10 μM)**		**Light (12.5 J/cm^2^) + RB (5 μM)**		**Light (20.0 J/cm^2^) + RB (5 μM)**		**Light (22.5 J/cm^2^) + RB (5 μM)**	

*A. baumannii* 128	GEN	6 (R)	≥256 (R)	6 (R)	≥256 (R)	6 (R)	≥256	6 (R)	≥256 (R)	6 (R)	128 (R)	6 (R)	64 (R)
	DOX (NR)	6.6	48	6.2	24	7.1	12	7.8	24	9.1	8	11.6	6
	SXT	6 (R)	≥32 (R)	6 (R)	≥32 (R)	6 (R)	≥32 (R)	6 (R)	≥32 (R)	6 (R)	≥32 (R)	6 (R)	≥32 (R)
	CIP	6 (R)	≥32 (R)	6 (R)	≥32 (R)	6 (R)	≥32	6 (R)	≥32 (R)	6.1 (R)	12 (R)	6 (R)	32 (R)
	IPM	7.3 (R)	≥32 (R)	8.3 (R)	≥32 (R)	8.2 (R)	12	8.9 (R)	≥32 (R)	9.8 (R)	8 (S/R)	14 (R)	2 (S)
	TZP	6	≥256	6	≥256	6	≥256	6	≥256	6.1	≥256	10.0	≥256
	CAZ (NR)	6	≥256	6	≥256	6	≥256	6	≥ 256	6	≥256	6	64
	SAM	8.0	48	9.2	44	7.4	16	8.8	32	9.1	24	14.8	12
	CST	13.7	0.094 (S)	15	0.19 (S)	13.6	0.094 (S)	14.1	0.094 (S)	15.9	0.094 (S)	16.9	0.094 (S)

*NR, not recommended; R, resistant; S, susceptible; CAZ, ceftazidime; CIP, ciprofloxacin; CST, colistin; DOX, doxycycline; GEN, gentamycin; IPM, imipenem; SAM, ampicillin-sulbactam; SXT, trimethoprim-sulfamethoxazole; TZP, piperacillin-tazobactam.*

**Table 3:** Sublethal and lethal aBL impact on *A. baumannii* drug susceptibility.

aBL (λ 411 nm)										
	**Antibiotic**	**Control**		**36.4 J/cm^2^**	**54.5 J/cm^2^**	**72.7 J/cm^2^**	**90.9 J/cm^2^**		**109.1 J/cm^2^**	
		**Disk diffusion [mm]**	***E*-test [μg/ml]**	**Disk diffusion [mm]**	**Disk diffusion [mm]**	**Disk diffusion [mm]**	**Disk diffusion**	***E*-test [μg/ml]**	**Disk diffusion [mm]**	***E*-test [μg/ml]**

*A. baumannii* 127	GEN	6 (R)	≥256 (R)	6 (R)	6 (R)	6 (R)	6 (R)	≥256 (R)	6 (R)	≥256 (R)
	DOX (NR)	6	128	6.5	6	6	6	64	6	48
	SXT	6 (R)	≥32 (R)	6 (R)	6 (R)	6 (R)	6 (R)	≥32 (R)	6 (R)	≥32 (R)
	CIP	6 (R)	≥32 (R)	6 (R)	6 (R)	6 (R)	6 (R)	≥32 (R)	6 (R)	≥32 (R)
	IPM	6.8 (R)	≥32 (R)	8.2	8.0	7.6	7.7	≥32	8.6 (R)	≥32 (R)
	TZP	6	≥256	6	6	6	6	≥256	6	≥256
	CAZ (NR)	6	≥256	6	6	6	6	≥256	6	≥256
	SAM	6.5	48	7.8	7.3	7.5	8.1	32	6	24
	CST	14.0	0.094 (S)	14.0	14.3	14.1	15.4	0.125 (S)	16	0.125 (S)
*A. baumannii* 128	GEN	6 (R)		≥256 (R)	6 (R)	6 (R)	6 (R)		6 (R)	
	DOX (NR)	6.6	48	6.1	7.8	7.8	7.1	256 (R)	6 (R)	256 (R)
	SXT	6 (R)	≥32 (R)	6 (R)	6 (R)	6 (R)	6 (R)	≥32	7.2	16
	CIP	6 (R)	≥32 (R)	6 (R)	6 (R)	6	6	≥32 (R)	6 (R)	≥32 (R)
	IPM	7.3 (R)	≥32 (R)	7.8 (R)	8.1 (R)	8.2 (R)	8.8 (R)	≥32	6 (R)	≥32 (R)
	TZP	6	≥256	6	6	6	6	≥32 (R)	9.1 (R)	≥32 (R)
	CAZ (NR)	6	≥256	6	6	6	6	≥256	6	≥256
	SAM	8.0	48	7.6	7.2	7.5	7.2	≥256	6	≥256
	CST	13.7	0.094 (S)	13.8	14.2	14.7	13.9	48	8.9	32

*NR, not recommended; R, resistant; S, susceptible; CAZ, ceftazidime; CIP, ciprofloxacin; CST, colistin; DOX, doxycycline; GEN, gentamycin; IPM, imipenem; SAM, ampicillin-sulbactam; SXT, trimethoprim-sulfamethoxazole; TZP, piperacillin-tazobactam.*

### Serial Dilution Methodology for Synergy Testing

**FIGURE 3:**
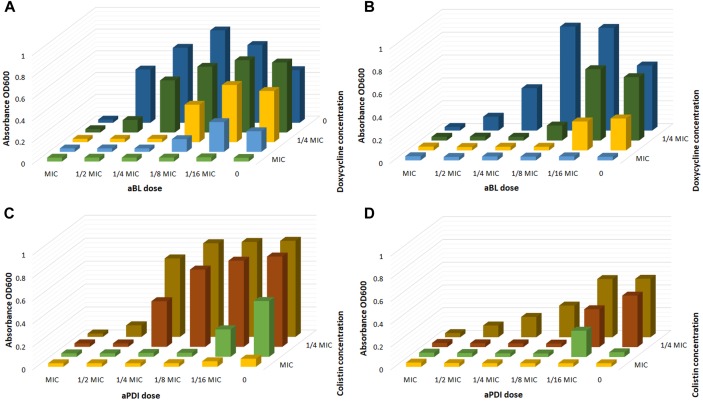
Checkerboard analysis. **(A)** Checkerboard analysis of aBL/doxycycline combined treatment for *A. baumannii* no. 128. **(B)** Checkerboard analysis of aBL/doxycycline treatment for *A. baumannii* no. 127. **(C)** Checkerboard analysis of aPDI/colistin combined treatment for *A. baumannii* no. 128. **(D)** Checkerboard analysis of aPDI/colistin treatment for *A. baumannii* no. 127.

To further confirm and/or detect other synergies, the serial dilution methodology was employed. Figure 3 exemplifies the checkerboard assay. The obtained results indicate that with the employment of phototreatment/antimicrobial combinations, successful *A. baumannii* eradication was achieved with the use of as little as ¼ of the MIC of doxycycline and colistin together with ¼ of the MIC of aBL and 1/8 of the MIC of aPDI (Figure 3).

Checkerboard FIC calculations confirmed the existence of synergistic interactions when phototreatment was used in combination with doxycycline, imipenem or colistin; furthermore, the results indicated a synergy between aBL and trimethoprim/sulfamethoxazole treatments (Table 4).

**Table 4:** Checkerboard FIC calculation.

Antibiotic	*A. baumannii* 127		*A. baumannii* 128	
	aBL	aPDI	aBL	aPDI
GEN	>0.5^a^	>0.5	>0.5	>0.5
DOX	**0.375**	>0.5	**0.5**	**0.375**
SXT	**0.5**	>0.5	>0.5	>0.5
CIP	>0.5	>0.5	>0.5	>0.5
IPM	>0.5	>0.5	>0.5	**0.375**
TZP	>0.5	>0.5	>0.5	>0.5
CAZ	>0.5	>0.5	>0.5	>0.5
SAM	>0.5	>0.5	>0.5	>0.5
CST	>0.5	**0.375**	>0.5	**0.375**

*Bold indicates possible synergistic interactions. ^a^FICI index; FICI = FIC_a_ + FIC_b_; CAZ, ceftazidime; CIP, ciprofloxacin; CST, colistin; DOX, doxycycline; GEN, gentamycin; IPM, imipenem; SAM, ampicillin-sulbactam; SXT, trimethoprim-sulfamethoxazole; TZP, piperacillin-tazobactam.*

**FIGURE 4:**
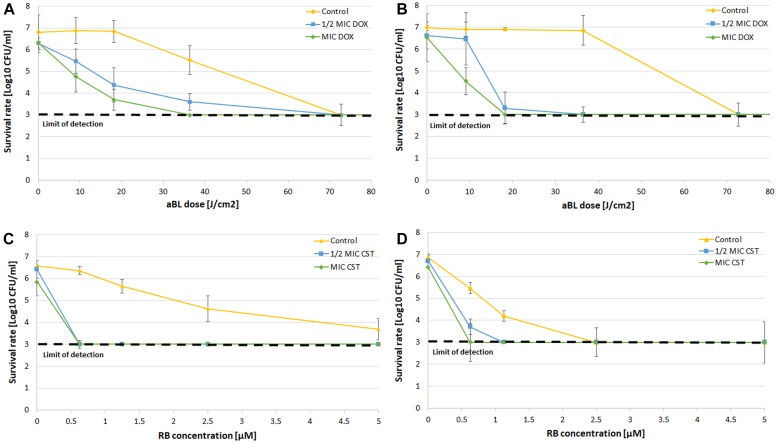
CFU counting after combined treatment. **(A)** Survival rate analysis of aBL/doxycycline combined treatment for *A. baumannii* no. 128. **(B)** Survival rate analysis of aBL/doxycycline treatment for *A. baumannii* no. 127. **(C)** Survival rate analysis of aPDI/colistin combined treatment for *A. baumannii* no. 128. **(D)** Survival rate analysis of aPDI/colistin treatment for *A. baumannii* no. 127. The detection limit was 1000 CFU/ml. The values are the means of three separate experiments. Control constituted of aBL or aPDI monotherapy, with no antibiotic administration. Values were combined by a line for better visualization of the data.

Along with checkerboard analysis, direct post-treatment probing and CFU counting were performed. The obtained results are exemplified by aBL/doxycycline and aPDI/colistin combinations (Figure 4). The obtained results clearly indicate that combined treatment led to more effective bacterial killing of both *A. baumannii* isolates with the employment of decreased antibiotic concentrations as well as lower aBL/aPDI doses (Figure 4). For all other combinations, the obtained results are summarized in Table 6, where all data from synergy testing via all included assays are shown. If characteristic “shifting” of bacterial survival rate curves was reported, the combination was marked with a “+” to indicate possible synergistic interaction.

### Time-Kill Curves for Synergy Testing

**FIGURE 5:**
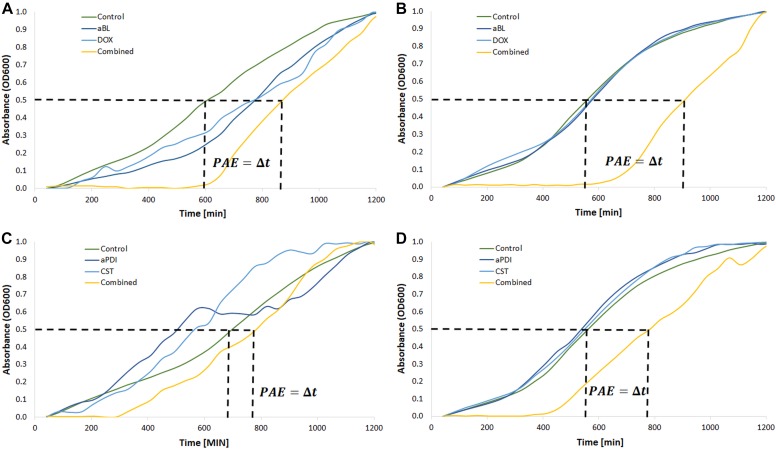
Postantibiotic effect testing. **(A)** Growth curve analysis of aBL/doxycycline combined treatment for *A. baumannii* no. 128. **(B)** Growth curve analysis of aBL/doxycycline treatment for *A. baumannii* no. 127. **(C)** Growth curve analysis of aPDI/colistin combined treatment for *A. baumannii* no. 128. **(D)** Growth curve analysis of aPDI/colistin treatment for *A. baumannii* no. 127. Phototreatments (aBL and aPDI) were employed with MIC doses. Antibiotics (doxycycline, DOX; colistin, CST) were administered at ½ MIC. Only one representative curve was presented.

Finally, possible synergies were confirmed and newly detected with PAE testing. The characteristic “shifting” of growth curves of *A. baumannii* pre-exposed with a combined treatment indicates that this approach delayed bacterial recovery (Figure 5). Figure 5 only show the results for aBL/doxycycline and aPDI/colistin combinations. In the case of *A. baumannii* no. 127, it is clear that only combined aBL/DOX and aPDI/CST treatment leads to delayed bacterial recovery resulting in PAEs of approximately 330 and 210 min, respectively (Figure 5B,D). In the case of *A. baumannii* no. 128, a clear indication of synergy was observed only for the aBL/DOX combination (Figure 5A); however, only a limited PAE was found for the aPDI and CST combined treatment (Figure 5C). This case exemplifies ambiguous results, which were marked “+/-” in the summary tables (Tables 5, 6). For all other combinations, the results are summarized in Tables 5, 6. If characteristic “shifting” of bacterial growth curves was reported, the combination was marked with “+” to indicate a possible synergistic interaction.

**Table 5:** Postantibiotic effect on *A. baumannii* clinical isolates.

Antibiotic	*A. baumannii* 127		*A. baumannii* 128	
	aBL	aPDI	aBL	aPDI
GEN	+	+	+/-	-
DOX	+	+	+	+
SXT	+	+	+/-	+/-
CIP	+	–	+/-	+/-
IPM	+	–	+	+/-
TZP	–	+/-	–	–
CAZ	–	+/-	–	–
SAM	+	+/–	+	–
CST	–	+	+	–

*(+) synergistic effect; (+/-) partial synergy; (–) no synergistic effect. CAZ, ceftazidime; CIP, ciprofloxacin; CST, colistin; DOX, doxycycline; GEN, gentamycin; IPM, imipenem; SAM, ampicillin-sulbactam; SXT, trimethoprim-sulfamethoxazole; TZP, piperacillin-tazobactam.*

**Table 6:** Summarized results of synergy testing.

Antibiotic	*A. baumannii* 127									
	aBL					aPDI				
	
	*E* – test	Disk diffusion	Checkerboard assay	Survival rate	Post antibiotic effect	*E*- test	Disk diffusion	Checkerboard assay	Survival rate	Post antibiotic effect
GEN	+	–	–	+/-	+	–	–	–	–	+
DOX	+	+	+	+	+	+	+	–	+	+
SXT	–	–	+	+	+	–	–	–	–	+
CIP	–	–	–	+	+	–	–	–	–	–
IPM	+	+	–	+/–	+	+	–	–	–	–
TZP	–	–	–	+	+	–	–	–	–	+/–
CAZ	–	–	–	+/–	–	–	–	–	–	+/–
SAM	+	+	–	–	+	–	+	–	–	+/–
CST	–	–	–	+	–	–	+	+	+	+
	***A. baumannii* 128**									
	
GEN	–	+	–	–	+/–	+	–	–	–	–
DOX	+	–	+	+	+	+	+	+	+	+
SXT	–	–	–	–	+/–	–	–	–	–	+/–
CIP	-	-	-	-	+/-	-	-	-	-	+/-
IPM	-	+	-	+	+	+	+	+	-	+/-
TZP	-	-	-	-	-	-	+	-	-	-
CAZ	-	-	-	-	-	+	-	-	-	-
SAM	+	-	-	+/-	+	+	+	-	+/-	-
CST	-	+	-	+	+	-	+	+	+	+/-

*CAZ, ceftazidime; CIP, ciprofloxacin; CST, colistin; DOX, doxycycline; GEN, gentamycin; IPM, imipenem; SAM, ampicillin-sulbactam; SXT, trimethoprim-sulfamethoxazole; TZP, piperacillin-tazobactam.*

The most pronounced indication concerned a aBL/aPDI and DOX combined treatment (Table 5). Clear “shifting” and a significant *Δt* (PAE) were observed for exposure to the combination of DOX and either aBL or aPDI. The same result was reported in the case of aBL/SAM and aBL/IPM combinations. Other inconclusive results were found for aBL treatment combined with GEN, SXT or CIP. In these cases, the indisputable results were observed for only one of two studied *A. baumannii* isolates. The same was observed for the aPDI and SXT combination. Significant strain dependence was reported for aBL/CST, aPDI/GEN and aPDI/CST combined treatments.

### Possible aBL/aPDI and Antimicrobials Synergies

All data collected within the current study are summarized in Table 6 to provide better insight into the possible synergies (Table 6). The most potent strain-independent synergies are marked with gray.

Some antimicrobials (i.e., DOX) interact synergistically with both aBL and aPDI treatments, and other interactions were observed when endogenously produced or exogenously administered PSs were involved in photodynamic inactivation (i.e., IPM and SAM with aBL treatment and CST with aPDI) (Table 6). Interestingly, some antimicrobials had synergies with phototreatments, but they were strain-dependent suggesting that no general conclusion concerning the possible synergy could be drawn (i.e., SXT and CST when combined with aBL for *A. baumannii* nos. 127 and 128, respectively, or IPM and SAM interacting synergistically with aPDI in case of *A. baumannii* no. 128) (Table 6). As expected, various synergy testing methods detected different synergistic interactions, indicating that the employment of various techniques is mandatory.

### Increased ROS Generation Could Explain the Mechanism Underlying the Observed Synergies

**FIGURE 6:**
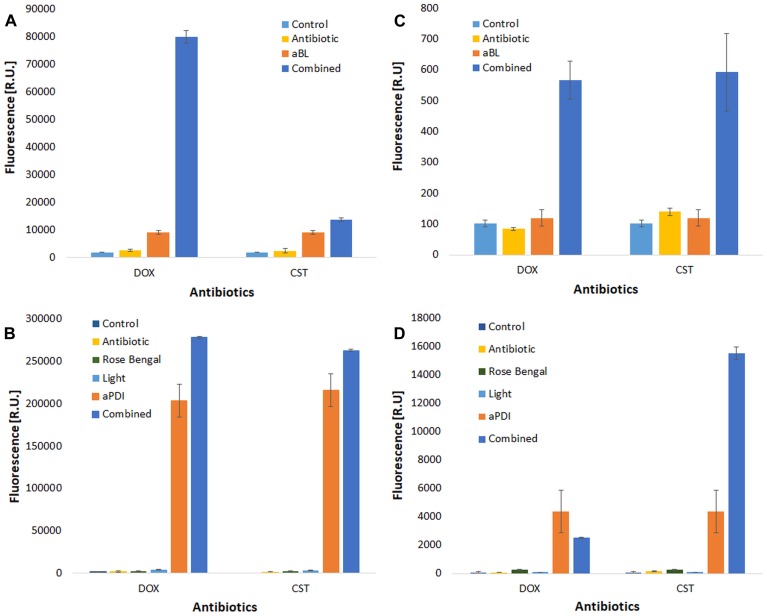
ROS detection. ROS detection was performed for two antimicrobials (doxycycline, DOX and colistin, CST). Cell-free suspensions of antimicrobials and/or rose bengal were incubated with ROS-detecting fluorescent probes to detect hydroxyl radicals (^∙^OH) [panels **(A)** aBL treatment, and **(B)** aPDI treatment] and singlet oxygen [**(C)** aBL treatment, and **(D)** aPDI treatment] upon irradiation. The values are the means of three separate experiments.

To investigate whether increased ROS production is responsible for the synergies between aBL/aPDI and antimicrobials, the level of ROS generated upon combined treatment was examined for four combined treatments (Figure 6).

As expected, effective ROS generation was observed upon only photodynamic treatment. Interestingly, the level of generated ROS was significantly increased when a combined treatment was employed (Figure 6). The most pronounced increase in ROS generation was reported for the aBL and DOX combination (Figure 6A), but a similar effect was observed for all studied combined approaches. This discovery supported one of several possible mechanisms underlying the observed synergies.

## Discussion

The most recent discoveries concerning combinations of aBL/aPDI and antibiotics indicate that photoinactivation sensitizes microorganisms to routinely used antimicrobials [most recently reviewed by Wozniak and Grinholc (2018)]. If it is confirmed with the employment of approved methodology and translated into *in vivo* and clinical applications, this approach could improve the clinical outcome of i.e., wound infections caused by MDR pathogens and might reduce usage of antibiotics in the long term. Only a limited number of methodologies are adequate for investigation of synergistic interactions between various antibacterial approaches. This recommendation explains the employment of all the indicated methods within the current study. Moreover, the current study is the first to describe aBL/aPDI interactions with antimicrobials covering all antibiotic categories as well as all antimicrobial mechanisms of action.

The significant bactericidal efficacy of both aBL and aPDI against *A. baumannii* was repeatedly reported in numerous published *in vitro* and *in vivo* studies (Dai et al., 2009; Huang et al., 2014; Maisch et al., 2014; Zhang et al., 2014; Yuan et al., 2017; Yang et al., 2018). However, the first published evidence of a combined aPDI/antibiotic approach being used against pandrug-resistant *A. baumannii* was presented by Boluki et al. (2017). Their research evidenced that aPDI affects the expression level of genes responsible for *Acinetobacter* resistance to colistin, i.e., *pmrA* and *pmrB*. In the case of other microbial species, the enhanced bacterial killing of the combined approach was frequently reported using *in vitro* planktonic (Almeida et al., 2014; Fila et al., 2017; Branco et al., 2018) biofilm (Perez-Laguna et al., 2017; Zhang et al., 2017) and *in vivo* models (Lu et al., 2010; Chibebe et al., 2013). The mentioned studies indicate that employing various culture media and experimental conditions, one could report different results; thus, it is mandatory to utilize standardized and approved methodology for synergy testing, which was the issue of prime importance within the current study.

The mechanisms underlying aBL/aPDI and antimicrobial interactions have never been identified, although some hypotheses have already been drawn (Wozniak and Grinholc, 2018). First, the synergistic effects may result from the increased permeability of the cell envelope resulting from aBL/aPDI-induced damage of this structure, which leads to increased antibiotic uptake into bacterial cells (Hewelt-Belka et al., 2016; Kossakowska-Zwierucho et al., 2016; Dai, 2017). Another possible mechanism could be the oxidative stress resulting from photochemical reactions and inhibiting the expression of genes determining microbial drug resistance (Boluki et al., 2017). Furthermore, the high bactericidal efficacy of combined approach can be explained by the fact that PSs can be substrates for efflux pumps. This competition between PSs and antimicrobials leads to increased uptake of antibiotics after the permeabilization of bacterial cell envelopes (Shih and Huang, 2011). In addition and most recently, He et al. (2018) published results confirming that some antimicrobials may express dual activity (He et al., 2018). They reported that tetracyclines may function as dual-action light-activated antibiotics expressing photosensitizing activity; this phenomenon may thus explain the synergy between aBL/aPDI and DOX within the current study. Another possible explanation for the combined treatment synergy could be concluded from the fact that both aBL/aPDI and antibiotic treatments lead to increased ROS production (Van Acker and Coenye, 2017); thus, phototherapy may lead to increased bactericidal efficacy and synergy via potentiation of the oxidative stress induced by antibiotic administration. It is only a hypothesis, as the mediation of ROS by antibiotic action has been an issue of concern in numerous literature studies. Within the current study, an effort was made to determine the role of ROS in the enhanced bacterial killing by combined treatments. The obtained data confirmed that increased ROS generation occur upon combined aBL/aPDI and antimicrobial treatment, indicating a possible explanation for the mechanism underlying this interaction.

## Conclusion

The described above issues indicate possible explanations for increased bactericidal efficacy of aBL/aPDI and antimicrobials when administered in combination; thus, the development of an alternative combined aBL/aPDI and antibiotics treatment seems to be justified and desired. The combined approach results not only in increased antimicrobial efficacy of but also decreased concentrations of antimicrobials, which may greatly slow the increasing rate of drug resistance (Dai, 2017).

## Ethics Statement

All isolated strains were collected during routine sampling. The current study only describes a collection of bacteria that comprised strains obtained from patients. Data collected from patients were anonymized and restricted to the information of the type of specimen and infection the strains were isolated from. Ethical approval was therefore not required. Moreover, the manuscript contains no data concerning animal studies, studies involving human subjects or inclusion of identifiable human data or clinical trials; thus, no ethical approval was required.

## Author Contributions

AW did the experimental work and participated in conception of the study. AR-Z performed studies concerning rose Bengal checkerboard analysis. NM participated in the data interpretation and critical manuscript review. MG has been involved in the coordination, conception, and design of the study and wrote the manuscript. All of the authors have read and approved the final manuscript.

## Conflict of Interest Statement

The authors declare that the research was conducted in the absence of any commercial or financial relationships that could be construed as a potential conflict of interest.

## Footnotes

1 https://aac.asm.org/
